# Polish norms for a set of colored drawings of 168 objects and 146 actions with predictors of naming performance

**DOI:** 10.3758/s13428-022-01923-3

**Published:** 2022-08-01

**Authors:** Agata Wolna, Magdalena Łuniewska, Ewa Haman, Zofia Wodniecka

**Affiliations:** 1 Institute of Psychology, Jagiellonian University, Kraków, Poland; 2 Faculty of Psychology, University of Warsaw, Warsaw, Poland

**Keywords:** Picture naming, Normative data, Polish, Object naming, Action naming, Name agreement, Age of acquisition, Image agreement, Imageability, Concept familiarity, Lexical frequency, Goodness of depiction, Word complexity

## Abstract

In this study, we present the first database of pictures and their corresponding psycholinguistic norms for Polish: the CLT database. In this norming study, we used the pictures from Cross-Linguistic Lexical Tasks (CLT): a set of colored drawings of 168 object and 146 actions. The CLT pictures were carefully created to provide a valid tool for multicultural comparisons. The pictures are accompanied by norms for Naming latencies, Name agreement, Goodness of depiction, Image agreement, Concept familiarity, Age of acquisition, Imageability, Lexical frequency, and Word complexity. We also report analyses of predictors of Naming latencies for pictures of objects and actions. Our results show that Name agreement, Concept familiarity, and Lexical frequency are significant predictors of Naming latencies for pictures of both objects and actions. Additionally, Age of acquisition significantly predicts Naming latencies of pictures of objects. The CLT database is freely available at osf.io/gp9qd. The full set of CLT pictures, including additional variants of pictures, is available on request at osf.io/y2cwr.

**Supplementary Information:**

The online version contains supplementary material available at 10.3758/s13428-022-01923-3.

## Introduction

Tasks which require participants to name pictures are widely used in many domains, including psychological, neurolinguistic, and logopedic diagnosis (e.g., Boston Naming Test: Kaplan et al., 1983; CAT: Fyndanis et al., 2017; Howard et al., 2010; CLTs: Haman et al., 2015; PPVT-4: Dunn & Whiteside, 2007; TDQ-60: Macoir et al., 2018), experimental research focused on memory (e.g., Brady et al., 2008; Weinstein & Shanks, 2010), visual perception (e.g., Grossman & Blake, 2002; Madan et al., 2018) and emotional processing (e.g., Lee et al., 2007; Schupp et al., 2004)^1^. In experimental psycholinguistics, naming latencies and picture-naming accuracy allow researchers to study the processes underlying speech production and language processing in general. Naming a picture aloud involves several stages, such as perceptual and conceptual identification of the object, selection of a suitable name, encoding the word-form (i.e., phonological, morphological, and phonetic representations) and articulation (Caramazza, 1997; Levelt et al., 1999; Shao, Roelofs, & Meyer, 2014). There are many factors that can influence the ease and speed of word retrieval and picture-naming accuracy; some are related to the perceptual and conceptual processing of the picture (e.g., the perceptual complexity of an image or how well it represents the depicted object; Bates et al., 2003; Dell’acqua et al., 2000); others are related to the lexical retrieval of the name itself (e.g., frequency of use of the picture’s name, its Age of acquisition etc.). As a consequence, the time needed to name a picture not only reflects the word retrieval and production process but is also affected by the characteristics of a given picture as well as the language spoken by the participant. As such, normed databases of pictures provide valuable information for experiments using pictorial stimuli in naming tasks. In the current paper, we provide psycholinguistic norms for a set of colored drawings of objects and actions derived from the Cross-Linguistic Lexical Tasks (LITMUS-CLTs, henceforth: CLT pictures; Haman et al., 2015, 2017), as well as their corresponding names in Polish, a Slavic language for which there was previously no normed database of pictures. We also report the predictive value of a set of predictors of *Naming latencies* for these pictures. From now on, we will refer to the norms collected for the CLT pictures as the CLT database.

### Norming studies of picture databases

A norming study of a picture database provides a set of measures which describe the characteristics of each picture in the set, including information on the dominant name and the characteristics of the pictures themselves, the concepts depicted in a picture, and the names of pictures^2^. In a seminal study, Snodgrass and Vanderwart (1980) provided normative data on a set of 260 black-and-white line drawings of objects. This original set has since been normed for different languages (Chinese: Weekes et al., 2007; Yoon et al., 2004; French: Alario et al., 2004; Alario & Ferrand, 1999; Icelandic: Pind et al., 2000; Japanese: Nishimoto et al., 2005; Spanish: Cuetos et al., 1999; Sanfeliu & Fernandez, 1996) and populations (norms for English-speaking children aged 8–10 years: Berman et al., 1989. English-speaking children aged 5–6 years: Cycowicz et al., 1997. Norms for an elderly Dutch population: Paesen & Leijten, 2019. Norms for aphasic patients: Cuetos, Aguado, Izura, & Ellis, 2002). A color version of the database was also normed for different languages (Chinese: Weekes et al., 2007, Greek: Dimitropoulou et al., 2009, French: Rossion & Pourtois, 2004, Kannada: Bangalore et al., 2022, Russian: Tsaparina et al., 2011, comparison of 14 European languages: Torrance et al., 2018). Over the last 20 years, other databases of pictures have been introduced and normed to provide either a more naturalistic alternative to Snodgrass and Vanderwart’s original database or to provide a larger database of pictures and norms for multiple languages. Examples of databases providing a set of ecologically valid pictures include the BOSS database, which consists of pictures based on real object photographs (Dutch: Decuyper et al., 2021; English: Brodeur et al., 2010; French: Brodeur et al., 2012; Portuguese: Souza et al., 2021; Thai: Clarke & Ludington, 2018); the LinguaPix database, which contains 1620 photographs of objects (German: Krautz & Keuleers, 2021); and the set of photos and real object-based images assembled by Moreno-Martinez and Montoro (initial study in Spanish: Moreno-Martínez & Montoro, 2012; Dutch: Shao & Stiegert, 2016; Portuguese: Souza et al., 2021). Large databases of pictures and norms for multiple languages are provided by, e.g., the MultiPic database, which contains 750 items normed for German, Italian, Spanish, English, French and Dutch (Duñabeitia et al., 2018); the International Picture Naming Project, which contains 520 pictures (including the original Snodgrass and Vanderwart database) normed for Bulgarian, Chinese, English, German, Hungarian, Italian, and Spanish (Bates et al., 2003; Székely et al., 2004; Székely et al., 2005); an extension of the International Picture Naming project, which contains 590 pictures (Belgian Dutch: Severens et al., 2005); the European Picture Pool for oral naming (PEDOI), which contains 390 line drawings normed for Canadian French, Dutch, English, French, German Italian, Spanish, Swedish and Russian (Kremin et al., 2003; Sirois et al., 2006); and the previously mentioned BOSS database with all its extensions, which consists of 1468 pictures (Brodeur et al., 2014).

While most of these databases contain images representing simple objects, there are a growing number of normed databases of pictures representing actions (Dutch: Shao et al., 2014; English: Fiez & Tranel, 1997; Székely et al., 2005; French: Bonin et al., 2004; German: Busch et al., 2021; Gulf Arabic: Khwaileh et al., 2018; Russian: Akinina et al., 2015; Spanish: Cuetos & Alija, 2003; Turkish: Bayram et al., 2017). So far, only three of these studies have provided norms and comparisons of pictures of both objects and actions (English: Székely et al., 2005; Gulf Arabic: Khwaileh et al., 2018; Turkish: Bayram et al., 2017). All of these studies were based on sets of black-and-white drawings which were either a combination of pictures from different databases and psycholinguistic tests (like the IPNP database, Székely et al., 2005) or a set of custom images created for the purposes of a given study (Bayram et al., 2017; Khwaileh et al., 2018).

### Typical picture and word characteristics in norming studies of picture databases

Normed databases of pictures typically contain a number of different indices that relate to the characteristics of the pictures. These can include (1) *Name agreement* indices (such as a measure of entropy (H) or Name agreement (%)) and (2) *Goodness of depiction* ratings. They can also include measures which refer to the concepts depicted in the pictures, such as (3) *Image agreement* or (4) *Concept familiarity*. Normed databases usually also include a number of indices that refer to the names of pictures in the given database, such as (5) *Age of acquisition* (6) *Imageability*, (7) a word’s lexical *Frequency*, and indices of a word’s (8) *Complexity*, such as length (measured in phonemes or syllables), number of morphemes, and information on the transitivity and reflexivity of verbs. Below we briefly discuss indices related to the characteristics of pictures and their corresponding names that were used in the current project.^3^

### Characteristics of pictures

1. *Name agreement* is a measure of the consensus between individuals when choosing a name for a given picture. Typically, it is obtained in a task in which participants name pictures of single objects or actions presented one by one. As shown by many previous normative studies, *Name agreement* is a very good predictor of *Naming latencies* that does not seem to be subject to substantial cross-linguistic differences. A study in British English (Barry et al., 1997) found that *Name agreement* influences the *Naming latencies* of pictures representing objects, with lower *Name agreement* corresponding to longer *Naming latencies*. This result has been replicated in American English (Székely et al., 2003), Belgian Dutch (Severens et al., 2005), Chinese (Liu et al., 2011), French (Alario et al., 2004), Gulf Arabic (Khwaileh et al., 2018), Italian (Dell’acqua et al., 2000; Navarrete et al., 2019), Kannada (Bangalore et al., 2022) and Spanish (Cuetos et al., 1999). Similar results have also been found for pictures depicting actions in Dutch (Shao et al., 2014), French (Bonin et al., 2004), German (Busch et al., 2021) and Spanish (Cuetos & Alija, 2003). *Name agreement* has also been shown to be a significant predictor of *Naming latencies* in a combined database of pictures of objects and actions (Gulf Arabic: Khwaileh et al., 2018; American English: Székely et al., 2005). According to the EEG study by Cheng et al. (2010), *Name agreement* affects two processes engaged in picture naming: object recognition and lexical selection. Following this interpretation, *Name agreement* should reflect either the ease of recognizing a picture or the lexical competition between the alternative names. However, norming studies of different databases have not found a consistent relationship between *Name agreement* and measures describing picture stimuli, like *Visual complexity* or *Goodness of depiction*, which should influence object recognition (Bates et al., 2003). Therefore, it seems that competition between alternative names might be a more plausible locus of the *Name agreement* effect.
2. *Goodness of depiction* reflects how well a depicted object or action matches its dominant name. Typically, it is obtained in a task in which participants provide subjective ratings of how well a presented picture or image corresponds to its provided (dominant) name. In a norming study on seven different languages, Bates et al. (2003) showed that higher *Goodness of depiction* scores were related to faster *Naming latencies*. Interestingly, the *Goodness of depiction* ratings collected in English were associated with substantially faster *Naming latencies* in every other language of the study (Bates et al., 2003). This suggests that *Goodness of depiction* may be insensitive to cross-linguistic differences and as such it may be related not to lexical processing but to picture recognition processes.
3. *Image agreement* is a measure corresponding to the conceptual representation of a depicted object or action. It refers to the degree to which the mental image of an object formed in response to its name corresponds to the presented picture. Typically, it is obtained in a task in which participants imagine an object or action referring to a presented word and rate how closely it resembles a picture which is presented a couple of seconds later. Higher *Image agreement* scores have been shown to be related to faster *Naming latencies* in several norming studies on different languages and databases of actions (Bangalore et al., 2022; Bonin et al., 2004; Schwitter et al., 2004; Shao et al., 2014) and objects (Alario et al., 2004; Cuetos et al., 1999; Torrance et al., 2018). However, not all norming studies, especially those concerning databases of objects, found *Image agreement* to be a significant predictor of naming latencies (Barry et al., 1997; Weekes et al., 2007). *Image agreement* is presumed to affect early picture-naming processes related to image recognition (Snodgrass & Vanderwart, 1980). Specifically, *Image agreement* is thought to reflect how closely a picture resembles the speaker’s mental image of a depicted object or action (Barry et al., 1997). As such, it has been argued to be a proxy for the presence of more canonical mental images for a depicted object or action (Akinina et al., 2015), which consequently makes the image recognition process easier and results in shorter *Naming latencies*. These inconsistencies in finding the relationship between *Image agreement* and *Naming latencies* in different norming studies might, on one hand, reflect cross-linguistic (and cross-cultural) differences in canonical mental images of given objects or actions; on the other hand, they might reflect differences between databases of pictures or photographs.
4. *Concept familiarity* refers to the extent to which a person comes into contact with a depicted object or action, i.e., when this person thinks about or deals with it. Typically, it is obtained in a task in which participants estimate how often they come in contact with an object or action (i.e., see, think about, or perform it) presented in a picture. This measure is thought to reflect the retrieval of the semantic representations corresponding to the picture’s name (Hirsh & Funnell, 1995). It is also thought to affect memory performance for pictures and words in experimental tasks (Snodgrass & Vanderwart, 1980). As such, a higher *Concept familiarity* score (meaning easier access and retrieval of semantic representations) should be related to shorter *Naming latencies* in a picture-naming task. This dependency has been shown by some norming studies (Bangalore et al., 2022; Cuetos et al., 1999; Nishimoto et al., 2005; Torrance et al., 2018; Weekes et al., 2007); however, other experiments failed to observe any effect of *Concept familiarity* on picture *Naming latencies* (Alario et al., 2004; Bonin et al., 2004; Cuetos & Alija, 2003; Schwitter et al., 2004; Shao et al., 2014).

### Characteristics of words

(5) *Age of acquisition* is an estimation of the age at which a word is learned. Such estimations may be subjective or objective. A subjective *Age of acquisition* is typically obtained in a task in which participants give the age at which they think they learned a given word. Objective estimations are obtained through analysis of existing developmental corpora (e.g., CHILDES: MacWhinney, 2014), or the CDI database: Fenson, 2007) or are based on spontaneous speech samples of children of various ages. In the latter case, *Age of acquisition* is defined as the age at which a given word appears in the speech of most of the studied children (or the percentage of children meeting an arbitrarily set criterion, see: Łuniewska et al., 2016). Independently of the type of *Age of acquisition* estimation (subjective vs objective)^4^, words acquired earlier in life are processed more quickly and accurately than words learned later (see Łuniewska et al., 2016 for review). *Age of acquisition* ratings are one of the most reliable predictors of picture *Naming latencies* across languages. The review provided by Bakthiar and colleagues (Bakhtiar et al., 2013: Table 1) shows that *Age of acquisition* was a significant predictor of *Naming latencies* across eight languages and across all studies included in this review. A Bayesian meta-analysis of 14 studies confirmed that *Age of acquisition* has a very strong influence on naming latencies across languages (Perret & Bonin, 2019), and it was the only significant predictor of *Naming latencies* in all studies included in this meta-analysis.
(6) *Imageability* is a measure of the ease with which a given name evokes mental images of a particular object or action (Alario et al., 2004). Typically, it is obtained in a task in which participants indicate how easy it is to elicit a mental image of an object or action corresponding to a given word. It is interpreted as a measure of the “richness” of semantic representations (Plaut & Shallice, 1993; Shao et al., 2014). According to this interpretation, names with high *Imageability* are easier to process at the semantic level; consequently, object recognition of pictures corresponding to highly imageable names is faster (Alario et al., 2004). Therefore, *Naming latencies* of pictures corresponding to highly imageable names should be faster than those corresponding to names of low imageability. This prediction has been confirmed by a number of norming studies (Alario et al., 2004; Shao et al., 2014); however, *Imageability* did not always turn out to be a significant predictor of *Naming latencies* (Bonin et al., 2004; Cuetos & Alija, 2003).
(7) *Frequency* reflects how often speakers of a particular language use a given word. It is a measure based on the analysis of large corpus of a given language (like movie subtitle analysis: Chinese – Cai & Brysbaert, 2010; Dutch – Keuleers et al., 2010; English – van Heuven et al., 2014; Polish – Mandera et al., 2015; Spanish – Cuetos, Glez-Nosti, Barbón, & Brysbaert, 2011). In picture-naming studies requiring a spoken response, *Frequency* has been shown to affect the naming process at the level of phonological processing (Jescheniak & Levelt, 1994). It has been observed that *Naming latencies* become shorter for higher *Frequency* names (Oldfield & Wingfield, 1965). While some norming studies have confirmed this finding (Alario et al., 2004; Barry et al., 1997; Bates et al., 2003; Cuetos et al., 1999; Torrance et al., 2018), others have failed to replicate it (Bangalore et al., 2022; Cuetos & Alija, 2003; Nishimoto et al., 2005; Schwitter et al., 2004; Severens et al., 2005; Székely et al., 2003; Weekes et al., 2007). Studies requiring a written response show a similar effect of *Frequency* to that observed in overt naming tasks: higher-frequency words are named faster than lower-frequency words (Baus et al., 2013; Bonin & Fayol, 2002; Qu, Zhang, & Damian, 2016; Zhang & Wang, 2014). Only two picture-norming studies tested the effects of *Frequency* on written naming latencies, but they found contradictory results (Bonin et al., 2004; Torrance et al., 2018). While the effects of *Frequency* on both spoken and written responses are thought to affect production at the lexicalization stage (Qu et al., 2016), the onset of the *Frequency* effect was found to be later in written production (Baus et al., 2013).
(8) *Complexity index* is a measure of a word’s phonological and morphological complexity. This measure was first introduced by Haman et al. (2015) and has been used as an index of word difficulty that is complementary to *Age of acquisition* in the development of CLTs. *Complexity index* is a composite measure which takes into account the number of a word’s characteristics that are related to its morphological and phonological properties; at the same time, it also takes into account indices related to exposure of speakers of a given language to a given word. Still, phonological aspects, such as word length in phonemes, have the strongest impact on the *Complexity index* score (see: Hansen et al., 2017; Van Wonderen & Unsworth, 2020). Although studies on word knowledge in monolingual (Haman et al., 2017) and bilingual children (Hansen et al., 2017; Van Wonderen & Unsworth, 2020) have shown that the *Complexity index* has no impact on performance in picture-naming and picture-recognition tasks in most languages, this measure has not yet been used in studies on adult timed picture naming.

### The current project

Here, we present the CLT database: a set of timed picture-naming norms in Polish, together with norms corresponding to the properties of these pictures, the concepts they represent, and words corresponding to the dominant and alternative names of these pictures (henceforth referred to as *names of pictures*).

The norms were collected in several steps. Norms for *Name agreement* and *Naming latencies* were collected in a laboratory setting in which participants overtly named all the pictures (we will further refer to this experiment as *the picture-naming study*). The norms for *Goodness of Depiction*, *Image agreement*, *Concept familiarity, Age of acquisition* and *Imageability* were collected in separate online experiments involving different groups of participants (we will further refer to those experiments as *studies on characteristics of pictures* in reference to *Goodness of depiction, Image agreement* and *Concept familiarity,* and *studies on characteristics of words* in reference to *Age of acquisition* and *Imageability*). *Frequency* estimates were obtained from the SUBTLEX-PL (Mandera et al., 2015) database, and *Word Complexity* was calculated based on the index proposed by Haman et al. (2015). The *Age of acquisition, Imageability, Frequency* and *Complexity index* values were obtained for all the dominant and alternative names of pictures used by the participants of the picture-naming study.

## Methods

### Stimuli

**Fig. 1:**
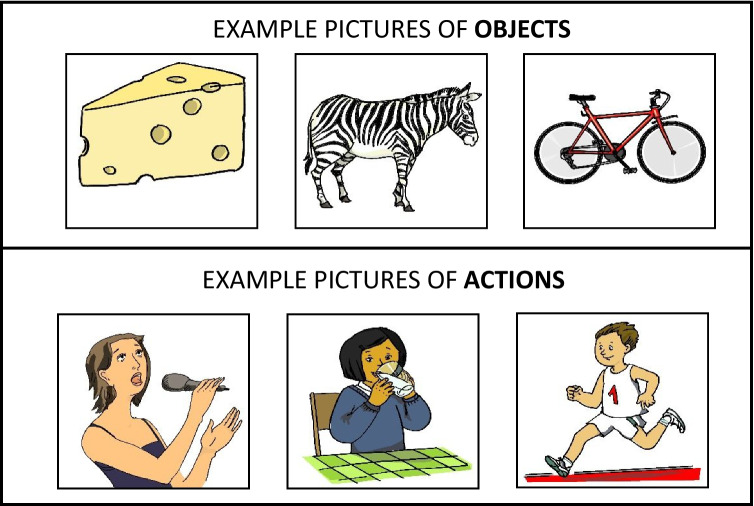
*Example CLT pictures representing objects and actions.* Note: the images above are for demonstration only and do not reflect how the pictures were presented to participants in the picture-naming study in the experimental procedure (where each picture was presented separately in an individual trial), nor how they are organized in the Cross-linguistic Lexical Tasks for children

A total of 168 pictures of objects and 146 pictures of actions were used in the study. The pictures used in the study come from a picture database originally designed for Cross-Linguistic Tasks (CLTs; Haman et al., 2015), which are a part of the LITMUS battery (Language Impairment Testing in Multilingual Settings; Armon-Lotem, de Jong, & Meir, 2015).^5^ Examples of pictures of objects and actions are presented in Fig. 1. The entire database of pictures is available on request at the following link: osf.io/y2cwr. This access limitation to the CLT pictures (i.e., they are shared only on request for research purposes) is dictated by the fact that they are a part of a battery of tests which is used to assess the comprehension and production of nouns and verbs in multilingual children. To ensure the validity of diagnoses based on CLTs, it is crucial to make sure the materials used in the tasks on are not freely available. For the same reason, using CLT pictures in research on children, especially multilingual ones, should be considered with caution because their results on Cross-Linguistic Tasks might not serve a diagnostic function if they are acquainted with the pictures.

### The picture-naming study

#### Participants

Ninety-eight participants (66 females, 32 males; Age: M = 22.48, SD = 3.11) took part in the picture-naming study, which was the basis for calculating norms for *Name agreement* and *Naming latencies*. For technical reasons, three participants were excluded from the analyses of pictures of objects. All participants were native Polish speakers who reported low proficiency in foreign languages (90 participants declared that they had had contact with or had learned more than one foreign language) and predominantly used Polish in their daily lives (for details, see Table 1). For the purpose of collecting data in a norming study of a picture database, testing participants who report low proficiency and late age of acquisition of their foreign languages is important as it has been shown that naming times in L1 are slower in speakers who know more than one language than in monolinguals (Ivanova & Costa, 2008). Hence, to reduce the effects of second-language proficiency on normed naming times, we only recruited participants who reported relatively low proficiency in their second language and whose knowledge of a foreign language corresponded to the levels attained during obligatory education at school. Participants received monetary remuneration or course credits in return for their participation. All studies reported in this paper met the requirements and gained the approval of the Jagiellonian University Institute of Psychology Research Ethics Committee or the Research Ethics Committee of the Faculty of Psychology at the University of Warsaw concerning experimental studies with human subjects.

**Table 1:** Information on the picture-naming study participants’ foreign-language experience. Values presented in the table correspond to mean ratings for each language. Standard deviations are given in parentheses

	L1	L2	L3^*^
	Onset of language acquisition (years)		
	native	11.13 *(4.63)*	15.04 *(3.73)*
	Self-reported proficiency		
Active use	8.72 *(3.73)*	4.77 *(2.02)*	3.02 *(2.17)*
Passive use	9.12 *(1.14)*	5.72 *(2.13)*	3.69 *(2.50)*
	Daily use		
Active use	95.23% *(7.27%)*	3.78% *(5.52%)*	1.24% *(4.08%)*
Passive use	82.99% *(13.10%)*	14.08% *(12.03%)*	3.27% *(6.60%)*

L1 – native language (Polish); L2 – second language in which participants have the highest proficiency; L3 – third language in which participants have the highest proficiency.

*90 participants reported that at some point in their lives they had started to learn L3.

*Onset of language acquisition* refers to the age at which a person started to learn a given language. *Proficiency* refers to a self-rated assessment of Active use (speaking) and passive use (understanding) of a given language on a scale from 1 to 10. *Daily use* refers to the percentage of active and passive use of a given language during an average day

#### Procedure

The picture-naming study was conducted in a laboratory. Participants completed the task in individual sound-proof testing booths. They were asked to name a set of 168 pictures of objects followed by a set of 146 pictures of actions which corresponds to the full set of CLT pictures used in this study. When naming the pictures of objects, they were instructed to answer the question “What is it?” (Polish: “Co to jest?”). When naming pictures of actions, they were instructed to answer the question “What is she / he doing?” (Polish: “Co ona / on robi?”). The instruction regarding how to name pictures appeared on the screen at the beginning of each part (pictures of objects / pictures of actions), and it was not displayed again before each picture. Pictures were presented using DMDX software (Forster & Forster, 2003). Each trial started with a fixation “+” point presented for 1000 ms in the center of a 24” screen (BenQ XL2411Z). Subsequently, a picture was presented in the same location. Pictures were presented in their original size (1654 x 1654 pixels) with no frame. The presentation time of each picture was adjusted to the participant’s response rate: after a response was detected, the picture remained on the screen for additional 3000 ms. If no response was registered, the picture disappeared 3000 ms after its display onset. Thus, we were able to ensure that we recorded the participants’ full answers, regardless of the individual response times. Participants’ responses were recorded with a condenser microphone (RØDE M3) placed in front of them on the table (such that it did not obscure the screen). Pictures of objects and actions were presented in separate sets: we did not mix the two types of pictures together in order not to impose additional task-switching-type demands that might influence the naming latencies. The order of presentation of the pictures was randomized within the sets of pictures of objects and actions; however, pictures of objects were always presented first, followed by pictures of actions. We used a fixed order of presentation for these two sets of pictures because naming pictures of actions is usually a more difficult task than naming pictures of objects, as indexed by longer naming times and lower name agreement (Bayram et al., 2017; Khwaileh et al., 2018; Székely et al., 2005). Using a fixed order of tasks allowed us to minimize the effect of preceding task difficulty**.** A short break was included between the object naming task and the action naming task.

### Series of studies on picture characteristics

#### Goodness of depiction

Thirty-six native Polish speakers took part in the *Goodness of depiction* study (26 females, age M = 19.58, SD = 0.95). They were asked to follow the instruction “Please rate the PICTURE in terms of whether it is an accurate EXAMPLE of the OBJECT depicted by the NOUN you used for it” by selecting one of the responses (0: “very good”; 1: “satisfactory”; 2: “a bit strange”; 3: “very strange”).^6^ (Polish: “Czy ten OBRAZEK jest trafnym przykładem obiektu, nazywanego przez podany przez ciebie RZECZOWNIK?” (0:” bardzo trafnym”; 1: “dosyć trafnym”; 2: “nieco dziwnym”; 3:” bardzo dziwnym”). The same response format was used in the section on verb assessment; the exact form of the instruction was: “It this PICTURE an accurate example of the action depicted by the VERB you used for it?” (Polish: “Czy ten OBRAZEK jest trafnym przykładem czynności, nazywanej przez podany przez ciebie CZASOWNIK?”). This instruction was preceded by a written picture-naming task. In the written picture-naming task, participants were asked to first assess whether the picture easily evoked a Polish noun/verb; if participants responded positively, they were asked to provide the first Polish word that came to mind as the name of the picture. Additionally, participants were asked to provide the English translation of the word (they were allowed to use dictionaries for this) in order to enable further cross-linguistic comparisons (the same procedure has been applied in several other studies that tested different languages). The pictures within a set (nouns or verbs) were presented in random order, and the order of the two sets was counterbalanced across participants. The *Goodness of depiction* score for each picture is the mean of all participants’ ratings.

#### Image agreement

Forty-six Polish native speakers took part in the *Image agreement* study (37 females, age M = 23.83, SD = 6.26). The participants were asked to judge how closely each picture matched the mental image corresponding to the name of the word or action describing the picture. Words used in this task corresponded to the most frequent names of a given picture, derived on the basis of the picture-naming study. Each trial started with the presentation of a word, which remained on the screen for 4000 ms. The participants were instructed to read the word and to imagine the object, person, or action to which it referred. Subsequently, a picture corresponding to the word appeared on the screen and the participants were asked to judge the degree to which it was similar to what they had imagined. They were asked to answer the following question: “To what extent does the picture presented below correspond to the mental image you had in mind after reading the word?” (Polish: “W jakim stopniu poniższy obrazek jest zgodny z wyobrażeniem, które miałaś / miałeś w głowie po przeczytaniu słowa”) using the 1–7 scale. The task was divided in two parts: in the first part, participants rated the Image agreement between 168 nouns and pictures representing objects; in the second part, they rated the Image agreement between 142 verbs and pictures representing actions.

#### Concept familiarity

Thirty-seven Polish native speakers participated in the *Concept familiarity* rating study (28 females, age M = 22.9, SD = 5.09). The participants were asked to judge how often they think about or have contact with the object or action presented on the picture. First, participants rated all the pictures of objects; then, they rated all the pictures of actions. Within each part (objects and actions), the order of presentation of pictures was randomized. In each trial, one picture appeared on the screen along with a scale. There was no time limit to provide a response. For pictures representing objects, participants were instructed to answer the following question: “How often do you have contact with or think about the thing presented in the picture?” (Polish: “Jak często masz kontakt z tym, co przedstawia obrazek, albo o tym myślisz?”). For the pictures representing actions, they were instructed to answer the following question: “How often do you perform, see others perform, or think about the action presented in the picture?” (Polish: “Jak często wykonujesz tę czynność, widzisz, jak inni ją wykonują, albo o niej myślisz?”). The participants were asked to use a scale from 1 (completely unfamiliar) to 7 (very familiar) to mark their response.

#### The series of studies on word characteristics

Below we present a series of studies on word characteristics corresponding to the dominant names of 314 pictures from the CLT database and to additional alternative names established based on the picture-naming study. Altogether, the CLT database contains data corresponding to 314 pictures and 1246 words corresponding to the dominant and alternative names of pictures of objects (432 words) and actions (814 words).

#### Age of acquisition

Fifty-eight Polish native speakers took part in the *Age of acquisition* rating study (46 females, age M = 26.49, SD = 8.49). The age of acquisition norms corresponding to 75% of dominant and alternative names of objects (213 words) and 92% of dominant and alternative names of pictures of actions (750 words) were based on ratings from an existing database for Polish language (Łuniewska et al., 2016). We collected additional data for missing words corresponding to the pictures’ dominant and alternative names obtained in the picture-naming study. We collected norms for the missing names of objects which were used by at least five participants of the main study and for the missing names of actions which were used by at least eight participants of the main study. Using this cut-off, we collected additional norms for 73 nouns and 66 verbs^7^. The procedure used for collection of the additional words was as close as possible to the original *Age of acquisition* data collection procedure that was previously applied in 32 languages (Łuniewska et al., 2016, 2019). Participants were asked to provide the age (in years) at which they had learned each of the words. The exact question was: “When did I learn this word?” (Polish: “Kiedy nauczyłem/łam się tego słowa?”). Participants’ task was to type a number from 1 (if a word was acquired at the age of one year) to 18 (if a word was acquired at the age of 18 years or more). The Age of acquisition of each word was defined as the age at which the participants were able to comprehend the word, even if they could not use, read, or write this word (Łuniewska et al., 2016, 2019).

#### Imageability

Fifty-four Polish native speakers took part in the *Imageability* rating study (46 females, age M = 27.73, SD = 7.96; no data on gender for three participants; no data on age for seven participants). Similarly to *Age of acquisition*, *Imageability* norms corresponding to 85% of dominant and alternative names of objects (240 words) and 92% of dominant and alternative names of pictures of actions (755 words) were derived from an existing database (Łuniewska et al., 2021). We conducted an additional experiment to collect missing data for the dominant and alternative names which were used to name pictures in the picture-naming study. We collected the norms for the missing names of objects which were used at least by five participants of the main study and for the missing names of actions which were used by at least eight participants of the main study. Based on these criteria, we collected norms for an additional 48 nouns and 62 verbs. Participants were asked to assess how easily a given word evokes a mental image. The exact instruction for nouns was: “For each noun, please estimate how easily this word evokes a mental image” (Polish: “Dla każdego rzeczownika prosimy określić, jak łatwo to słowo wywołuje wyobrażenie”). The participants were asked to use a scale from 1 (not imaginable) to 7 (easily imaginable). The instruction for verbs was as follows: “For each verb, please estimate how easily this word evokes a mental image” (Polish: “Dla każdego czasownika prosimy określić, jak łatwo to słowo wywołuje wyobrażenie”). Within each set (nouns and verbs), the words were presented in random order, and the order of the two sets was counterbalanced across participants.

### Scoring

#### The picture-naming study

Participants’ responses in the picture-naming study were manually coded and *Naming latencies* were set manually for each answer. The naming latencies were determined based on a visual analysis of spectrograms of the recordings of individual responses to each picture. Naming latencies determined this way were then validated by listening to the audio recording preceding and following the determined response time of a given answer; if the response onset was clearly audible and no voice was heard in the part of the recording preceding the response time, it was considered correct.^8^ To determine the dominant response for each picture, we followed a procedure adapted by Bates et al. (2003). In the first step, we error-coded the responses as either *Valid response*, *Invalid response,* or *No response*.

*Valid responses* correspond to all the responses with a codable (audible and recognizable) name and a valid response time (no hesitations, false starts, etc.). All words articulated completely or to the point at which it was possible to recognize the answer were kept for further analyses if they were existing Polish words. In the case of verbs, we considered as valid all responses in which participants committed inflectional errors (e.g., “ora” instead of “orze” in the case of the verb “orać” – to plow; a total of 0.72% of valid responses), used the incorrect tense (0.1% of valid responses) or grammatical number (0.49% of valid responses), or used the reflexive verb incorrectly (by either adding the reflexive pronoun or skipping it; 2.14% of valid responses). In the case of pictures of objects, valid responses corresponded to 98.36% of all trials; in the case of pictures of actions, valid responses corresponded to 95.61% of all trials.

*Invalid responses* refer to responses with missing or invalid *Naming latencies*. We also considered neologisms as invalid if they did not share a root with an existing word (however, these cases were very rare: only 14 responses for verbs were discarded, which represents 0.1% of data for pictures of actions). In the case of pictures of objects, 0.32% of responses in all trials were invalid; in the case of pictures of actions, 0.87% of responses in all trials were invalid.

*No response* refers to cases in which participants provided no audible name for a picture, or they provided an incomplete name which could not be recognized. In the case of pictures of objects, no response was recorded for 1.21% of trials; in the case of pictures of actions, no response was recorded in 3.51% of all trials.

The norms reported in the current study, as well as all statistical analyses, were based only on valid responses: both invalid and no-response trials were excluded prior to statistical analysis. Following the second response-coding step described by Bates et al. (2003), all valid responses were coded according to the *Lexical Code* categories: *Lexical Code 1* corresponds to the dominant response; *Lexical Code 2* corresponds to any morphological modifications of the dominant response, i.e., variations that share a root or a key part of the word without changing its meaning; *Lexical Code 3* corresponds to the dominant name’s synonyms; *Lexical Code 4* corresponds to all answers that do not fall in any of the previous categories. The percent of answers falling into each of the four Lexical Code categories for each picture is available in an Excel file, which also contains the norms for all pictures and their corresponding norms on the indices described in this study. This Excel file can be found in the Supplementary Materials and in the repository on osf.io/gp9qd.

#### Studies on picture and word characteristics

*Name agreement* was calculated for each picture based on the valid responses. One way of indexing *Name agreement* is by calculating the percentage of answers matching the dominant response; however, this method is not sensitive to the overall number and the proportion of use of the alternative names. As a consequence, it underestimates the real *Name agreement* in cases in which only two names were used for a given picture but with a similar frequency. Therefore, a second index, *H,* corresponding to the information entropy (Shannon, 1948) was proposed as a more appropriate measure of *Name agreement* (Snodgrass & Vanderwart, 1980). In the picture-naming task, the entropy reflects the degree of uncertainty in naming pictures. It is calculated using the following formula:

$$H=\sum\nolimits_{i=1}^k{p}_i{\mathit{\log}}_2\left(\frac{1}{p_i}\right)$$

where *k* refers to the number of different names given to each object, and *p*_*i*_ is the proportion of participants giving each name. If all participants provide the same name for a given picture, its *H* is equal to zero. In the description of the CLT database, we provide information on both estimates of Name agreement: the Name agreement (%) and *H*.^9^ Additionally, to account for the variability in participants’ responses due to the rich derivational morphology of Polish, Name agreement for the action pictures was calculated in three different ways:

1. Based on the actual answers given by the participants, each morphological variation counted as a separate alternative name.
2. Based on answers recoded to the infinitive, which made it possible to unify the morphological variability in participants’ responses due to grammatical number or tense.
3. Based on unified infinitives, which count the different aspects of verbs and the reflexive and non-reflexive forms of verbs as one answer. In the case of aspect, the most frequent response was used as the target form; in the case of reflexive verbs, the form correctly describing the action in the picture was considered the dominant one.^10^

In the statistical analyses of pictures representing actions, the third strategy was used, according to which *Name agreement* was based on the most unified forms; however, all three *Name agreement* scores are available in the Supplementary Materials and at osf.io/gp9qd.

The estimates for *Frequency* were derived from SUBTLEX-PL (Mandera et al., 2015). Each most frequent name and all the alternative names used in the main study were assigned a corresponding lemma frequency estimate. However, SUBTLEX-PL can only derive *Frequency* estimates for individual words. In consequence, we were not able to obtain *Frequency* estimates for names of objects which were composed of more than one word (e.g., “wózek dla dzieci” (stroller for children)) or names of actions which required a complement (e.g., “brać prysznic” (to take a shower)) and all reflexive verbs, which in Polish always comprise the conjugated verb + the reflexive pronoun (“się”). Moreover, SUBTLEX-PL did not provide *Frequency* estimates for all names provided by the participants of the picture-naming study. As a consequence, we were not able to obtain *Frequency* estimates for 8.10% of all dominant and alternative names of objects and for 17.45% of all dominant and alternative names of actions.

*Complexity index* was calculated individually for each response as a composite measure consisting of phonological and morphological aspects of a given word, as well as exposure to the depicted object or action (see Table 2). To exemplify the *Complexity index* calculation, the noun “auto” (“car”) has a *Complexity index* of 0.106. This word has four phonemes and a normed length of – 0.947. There are no consonant clusters or initial fricatives (0 points); this word has no prefixes or suffixes and has only one stem (1 point). Cars were assessed as very often accessible to preschool children in Poland (0 points). The word “auto” is, however, a loanword (1 point).

$$\mathrm{CI}\left(\mathrm{auto}\right)={2}^{\ast }\ \left(-0.947\right)+1\ \left(\mathrm{one}\ \mathrm{stem}\right)+1\ \left(\mathrm{loanword}\right)=0.106.$$

**Table 2:** Calculations of the *Complexity index*. Table adapted form Hansen et al., 2017, and Van Wonderen & Unsworth, 2020

Measure		Contribution to CI
Phonology	Word length in phonemes	$2\times \frac{word\ length-{mean}_{word\ class}}{SD_{word\ class}}$
	Word initial fricative or affricate?	Yes = 1 point
	Word initial consonant cluster?	Yes = 1 point
	Word medial consonant clusters?	Yes = 1 point
Morphology	How many stems?	1 point per stem
	Is this word a derivation?	Yes = 1 point
	Is there a prefix or suffix added to the stem?	Yes, both = 2 points Yes, either = 1 point
Exposure	Is this object/action available to direct experience in your country?	No = 1 point
	How often would preschool children in your country have access to this object/activity?	Not at all / rarely = 1 point Quite often = ${~}^{1}\!\left/ \!{~}_{2}\right.$ point Very often = 0 points
	Is this word a loanword?	Yes = 1 point

On the other hand, the noun “ślizgawka” has a *Complexity index* of 8.638. This word has nine phonemes (normed length of 0.819); it has an initial frication (“ś”; 1 point), an initial consonant cluster (“śl”; 1 point), and internal consonant clusters (“zg” and “wk”; 1 point). This word has one stem (1 point) but it is a derivation (of the word “ślizgać”, Eng. “to slide”; 1 point) which was created by adding a suffix (“-ka”) to the stem (1 point). This object is rarely accessed by preschool children in Poland (1 point) and it is not a recent loanword (0 points).

$$\mathrm{CI}\left(\acute{s} \mathrm{lizgawka}\right)=2\;^\ast\;0.819+3\;\left(\mathrm{phonology}\right)+3\;\left(\mathrm{morphology}\right)+1\;\left(\mathrm{exposure}\right)=8.638$$

### Data analysis

For the correlational analyses, we used data aggregated by picture and answer (i.e., corresponding to the dominant and all alternative answers provided for a given picture). As such, our correlation matrix reflects the relationships between a picture’s characteristics and all its dominant and alternative names. For the pictures of verbs, we used the *Name agreement* estimates calculated based on unified infinitives (for details, see Methods*–* 3^rd^ way of calculating the *Name agreement* proposed for verbs). As the distribution of most of our variables was highly skewed, the correlation analysis was based on the Spearman’s rank correlation coefficient, which is robust to both non-normal distributions and outliers. The statistical significance of the correlations’ results was corrected for multiple comparisons using the Bonferroni correction.

For the analyses of predictors of *Naming latencies*, we used linear mixed-effects models (Bates et al., 2014). The analyses were conducted using the lmerTest package in R (version: 3.1.3; Kuznetsova et al., 2017). Even though this method of analysis was not widely used in previous norming studies on pictures (with the notable exceptions of recent experiments by Busch et al., 2021; Navarrete et al., 2019; Torrance et al., 2018), we chose to use linear mixed-effect models instead of multiple regression because mixed models allow better control over different sources of variance. In our analyses, we modelled three different sources of random variability: participants, pictures, and the alternative names for each of the pictures. Because our analysis was based on a database which included not only dominant names of pictures but also all alternative names provided by participants, using linear mixed-effect models instead of multiple regression analysis allowed us to separately and more accurately model the random variability related to the estimates of pictures and words. Prior to running the analyses, correlations between predictors were assessed using the Variance Inflation Factor (Fox, 2015), which identified a high degree of multicollinearity between the two *Name agreement* indices: %NA and entropy (*H*) (for pictures of objects: VIF_%NA_ = 11.26, VIF_*H*_ *=* 10.66; for pictures of actions: VIF_%NA_ = 3.26, VIF_*H*_ *=* 3.42^11^). Therefore, for further analyses we only used the entropy (*H*) to explore the effects of Name agreement as it is considered a more nuanced measure than %NA (see the paragraph on Name agreement in the Scoring section). The dependent variable, *Naming latencies*, was transformed using a reciprocal transformation (– 1000/RT) due to the right-skewed distribution of the data. Additionally, to normalize the distributions of the predictors, we transformed all highly skewed predictors using an inverse transformation (1/x for positively skewed data and 1/max(x)-x for negatively skewed data), and we transformed moderately skewed predictors using a square root transformation. Highly skewed predictors included entropy (*H*) *, Age of acquisition, Imageability,* and *Image agreement* for pictures of objects, and *Age of acquisition, Imageability,* and *Image Agreement* for pictures of actions. Moderately skewed predictors included *Complexity Index* for pictures of objects and entropy (*H*), and *Complexity Index* for pictures of actions. Finally, *Frequency* estimates were transformed to a logarithmic scale. All the continuous predictors were demeaned prior to running the mixed-model analysis. Separate models were fitted for object and action pictures^12^. In both cases, we first fitted a maximal model and then identified the best random effects structure following the recommendations of Bates et al. (2018).

Due to the non-normal distribution of most of the predictors, direct comparison between the norms and estimates corresponding to pictures of objects and actions were performed using a two-sample, two-sided Wilcoxon rank sum test (equivalent to a Mann–Whitney *U* test). The test was implemented with the wilcox_test() function, and the corresponding effect sizes (r) were computed using the wilcox_effsize() function, both of which are from the rstatix package in R (Kassambara, 2021). To account for multiple comparisons, we adjusted the significance threshold using the Bonferroni’s correction: as a result, *p* values < 0.005 were considered significant. Additionally, as *Complexity index* estimates are based on scales that are normalized within the set of words that they refer to (i.e., separately for names of objects and actions), prior to the analyses of differences between pictures of objects and actions, *Complexity index* estimates were normalized to allow direct comparisons between nouns and verbs. The scripts and data necessary to reproduce the analyses and figures presented in this paper are available at osf.io/gp9qd.

### Results

#### Descriptive statistics

**Fig. 2:**
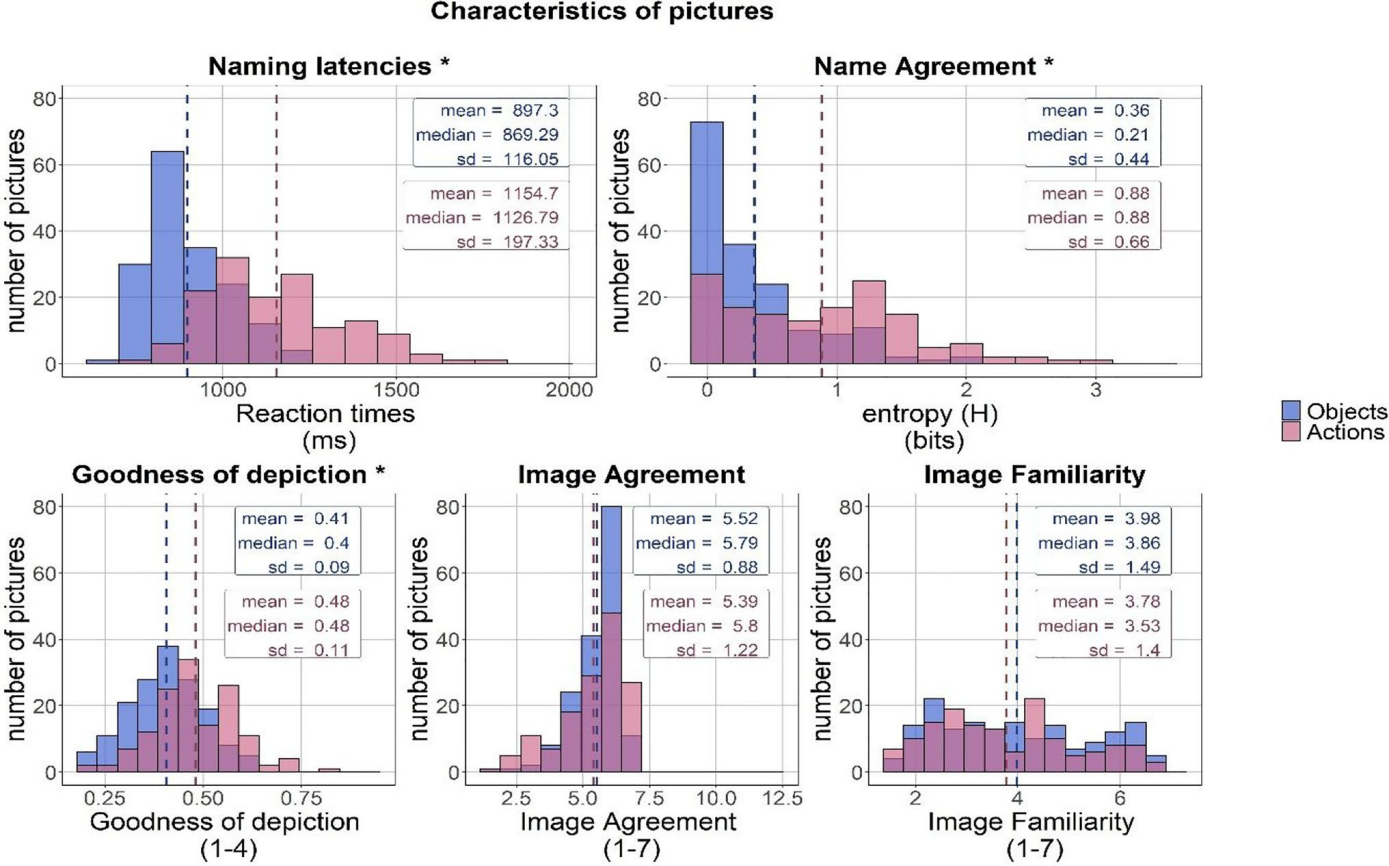
**Distribution of characteristics of pictures.** Note: The scale for each of the indices is given in parentheses. Binned data is represented by the histograms. *Dashed lines* correspond to mean scores for pictures of objects (*blue*) and actions (*red*). Note that for Goodness of depiction, lower values correspond to better scores. Names of variables for which significant differences were found in the two-sided Wilcoxon rank-sum test between pictures of objects and actions are marked with an *asterisk*. Note that this figure presents the distribution of the raw data prior to the transformations that normalized the data distribution that were necessary for statistical analyses

**Fig. 3:**
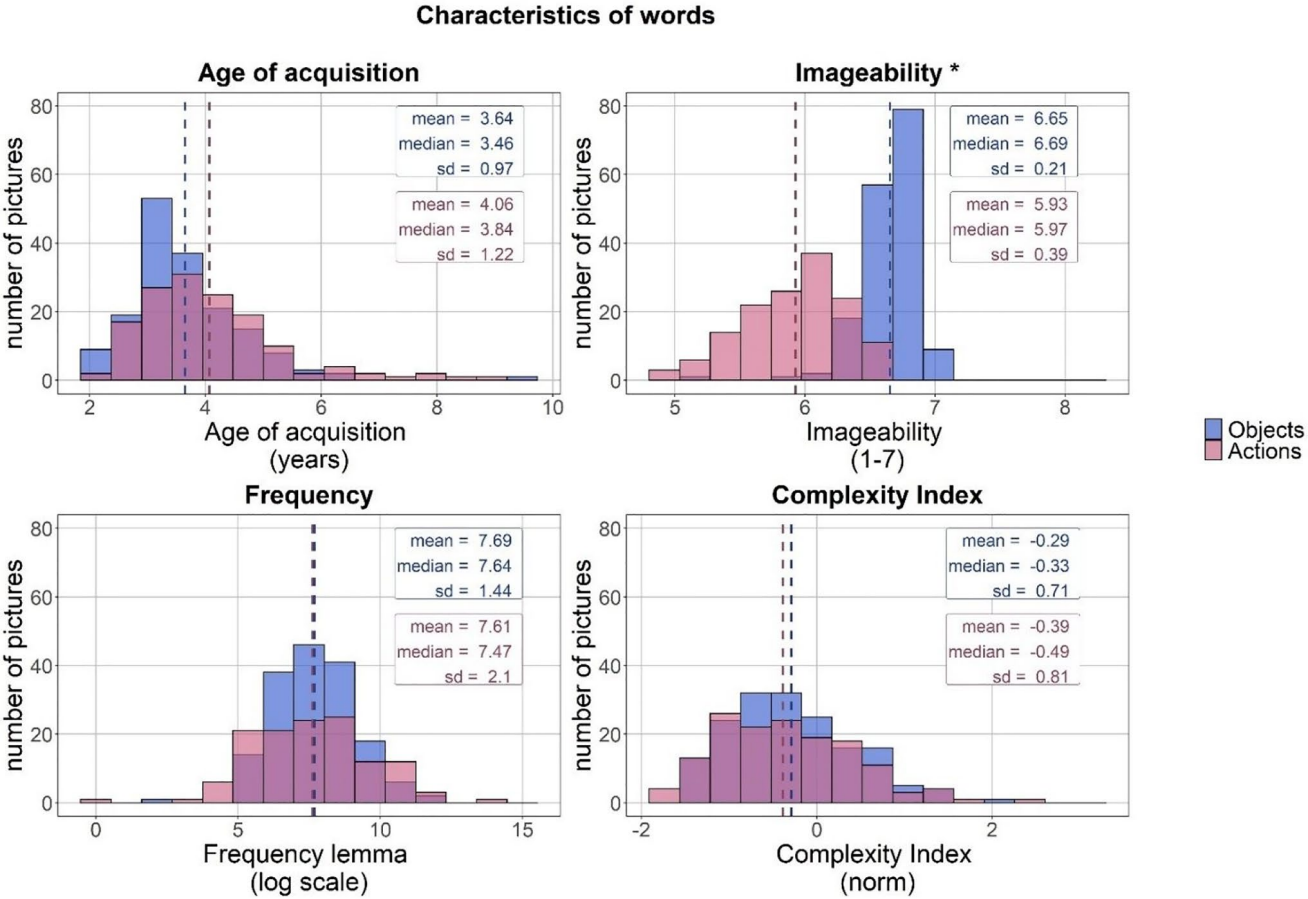
**Distribution of characteristics of words corresponding to the dominant names of pictures.** Note: The scale for each of the indices is given in parentheses. Binned data is represented by the histograms. *Dashed lines* correspond to mean scores for pictures of objects (*blue*) and actions (*red*). Names of variables for which significant differences were found in a two-sided Wilcoxon rank-sum test between characteristics of words corresponding to names of pictures of objects and actions are marked with an *asterisk*. Note that this figure presents the distribution of the raw data prior to the transformations that normalized the data distribution that were necessary for statistical analyses

Descriptive statistics corresponding to the variables derived from the norming study, namely entropy (*H*), *Name agreement (%)* and *Naming latencies*, are presented in Table 3. Furthermore, descriptive statistics for each of the variables corresponding to the properties of the pictures are presented in Table 4. Distribution of indices corresponding to the characteristics of pictures and their names are presented in Figs. 2 and 3, respectively). The complete norms for all the pictures are available in Excel files at osf.io/gp9qd as well as in the Supplementary Materials. Complete norms include two different summaries that are available for both pictures of objects and pictures of actions: the first contains a summary which only takes into account the dominant names of pictures; the second summary encompasses both the dominant and alternative names of pictures used by participants in the norming study. As norms for *Imageability* and *Age of acquisition* and estimates for *Complexity index* and *Frequency* were collected or calculated for a large portion of the alternative answers alongside the dominant names of pictures, the extended summary provides additional information on the characteristics of the alternative names of the CLT pictures.

**Table 3:** Descriptive statistics for norms derived from the main norming study: H statistic, Name agreement (%) and mean Naming Latency (ms) for pictures representing objects and actions

	Pictures of objects				Pictures of actions			
	*Mean*	*SD*	*Median*	*Range*	*Mean*	*SD*	*Median*	*Range*
*H*	0.36	0.44	0.21	0.00–1.92	0.89	0.66	0.9	0.00–2.94
*Name agreement (%)*	91.43%	13.18%	96.81%	32.63–100%	79.39%	18.68%	84.21%	26.32–100%
*Naming Latency (ms)*	897.33	116.74	867.46	692.67–1224.89	1157.70	197.33	1126.78	792.42–1795.67

The statistics were calculated based on the norms for the dominant name of each picture in the CLT picture database. For the pictures of actions, data corresponding to the unified infinitives were used

**Table 4:** Descriptive statistics for norms derived from our studies of the CLT database, broken down into pictures of objects and pictures of actions and their corresponding names

		Pictures of **objects**				Pictures of **actions**			
		*Mean*	*SD*	*Median*	*Range*	*Mean*	*SD*	*Median*	*Range*
*Goodness of depiction*		0.41	0.09	0.40	0.18–0.63	0.48	0.11	0.48	0.18–0.83
*Image agreement*		5.52	0.88	5.79	1.37–6.76	5.39	1.21	5.80	1.42–6.80
*Concept familiarity*		3.98	1.49	3.86	1.62–6.84	3.78	1.40	3.53	1.48–6.70
*Age of acquisition*		3.64	0.97	3.46	2.18–9.45	4.06	1.22	3.84	2.13–9.02
*Imageability*		6.65	0.21	6.69	5.22–6.98	5.93	0.39	5.98	4.82–6.63
*Frequency (log)*		7.69	1.46	7.64	2.20–11.94	7.61	2.10	7.47	3.64–14.42
*Complexity index*		– 0.29	0.71	– 0.33	– 1.51–1.20	– 0.39	0.81	– 0.49	– 1.86–2.55

The statistics were calculated based on the aggregated norms for each picture in the CLT database and the estimates for the corresponding dominant names. Goodness of depiction was rated on a scale from 0 to 4 (note that in this case a lower score corresponds to a higher Goodness of depiction rating). Image agreement, Image Familiarity and Imageability were rated on a scale from 1 to 7. Estimates for the Complexity index are presented on a normalized scale

#### Direct comparisons between pictures of objects and actions

Direct comparisons between characteristics of pictures of objects and actions revealed that the two sets of pictures were characterized by a similar *Age of acquisition* (W = 2157.5, *p* = 0.308, effect size *r* = 0.08), *Frequency* (W = 4824, *p* = 0.989, effect size *r* < 0.01), *Word Complexity* (W = 11,045, *p* = 0.734, effect size *r* = 0.02), and *Image Agreement* (W = 12012, *p* = 0.759, effect size *r* = 0.02). Differences between pictures of objects and actions were found for *Naming latencies* (W = 2848, *p* < 0.001, effect size *r* = 0.66), *Name agreement* (indexed by *H:* W = 6470.5, *p* < 0.001, effect size *r* = 0.41 and Name agreement (%): W = 17,674, *p* < 0.001, effect size *r* = 0.38), *Imageability* (W = 4437, *p* < 0.001, effect size *r* = 0.71), and *Goodness of depiction* (W = 6759, *p* < 0.001, effect size *r* = 0.36).

#### Correlation analysis

Full results of the correlation analysis between the *Naming latencies* and the variables corresponding to the properties of the pictures are presented in Table 5. In line with previous studies, our results revealed strong correlations between *Name agreement* (*H*) and *Naming latencies* for pictures of objects (*rho =* 0.56, *p* < 0.001) and actions (*rho =* 0.72 *p* < 0.001). Moderately strong correlations with *Naming latencies* were also found for *Concept familiarity* (*rho =* – 0.34, *p* < 0.001) and *Age of acquisition* (pictures of objects: *rho =* 0.32, *p* < 0.001; pictures of actions: *rho =* 0.28, *p* < 0.001). We also found a strong and negative correlation between the two *Name agreement* indices: entropy (*H*) and Name agreement (%) (pictures of objects: *rho =* – 0.94, *p* < 0.001; picture of actions: *rho* = – 0.84, *p* < 0.001). Moreover, both *Name agreement* indices showed a significant correlation with *Image agreement* (*H:* names of objects: *rho* = – 0.42, *p* < 0.001; names of actions: *rho* = – 0.31, *p* < 0.001; *Name agreement (%):* names of objects: *rho* = – .32, *p* < 0.001; names of actions: *rho* = – .21, *p* < 0.001) and *Complexity index* (*H:* names of objects: *rho* = – .29, *p* < 0.001; names of actions: *rho* = – .14, *p* < 0.001; *Name agreement (%)*: names of objects: *rho* = – 0.28, *p* < 0.001).

**Table 5:** Matrix of correlations between Naming latencies and variables corresponding to the characteristics of CLT pictures representing objects and actions and their names

	% NA	*H*	GoD	IA	Fam	CI	Imag	AoA	Freq
	Pictures of **objects**								
Naming latency	– 0.41*	0.56*	0.01	– 0.20*	– 0.34*	.18*	– 0.23*	0.32*	– 0.28*
Name agreement (%)		– 0.94*	0.04	0.32*	– 0.05	– 0.28*	0.09	– 0.13	0.24*
*H*			– 0.02	– 0.42*	– 0.04	0.29*	– 0.14	0.18*	– 0.27*
Goodness of depiction				– 0.07	0.06	– 0.04	– 0.04	– 0.07	0.12
Image agreement					– 0.07	– 0.13	0.11	– 0.05	0
Concept familiarity						– 0.07	0.23*	– 0.33*	0.19*
Complexity index							– 0.08	0.21*	– 0.44*
Imageability								– 0.75*	0.13
Age of acquisition									– 0.39*
	Pictures of **actions**								
Naming latency	– 0.55*	0.72*	0.01	– 0.32*	– 0.34*	0.12	– 0.06	0.28*	– 0.1
Name agreement (%)		– 0.84*	0.05	0.21*	0.13*	– 0.12	– 0.07	– 0.25*	0.05
*H*			– 0.09	– 0.31*	– 0.14*	0.14*	– 0.02	0.24*	– 0.12
Goodness of depiction				0.14*	0.04	– 0.01	0.06	0	– 0.01
Image agreement					0.17*	– 0.03	0.09	– 0.17*	0.01
Concept familiarity						– 0.1	0.2*	– 0.24*	0
Complexity index							– 0.08	0.32*	– 0.44*
Imageability								– 0.15	0.23*
Age of acquisition									– 0.54*

The values presented in the table correspond to Spearman’s rho correlation coefficients. Significance is marked with *asterisks.*

* *p* < 0.001 (significant results corrected for multiple comparison using Bonferroni correction)

%NA –Name agreement (%); GoD – *Goodness of depiction*; IA – Image agreement; Fam – Concept familiarity; CI – Complexity index; Imag – Imageability; AoA – Age of acquisition; Freq – Lemmatized frequency (log scale)

*Age of acquisition* was significantly and negatively correlated with *Imageability* for the names of objects (*rho =* – 0.75, *p* < 0.001)*, Concept familiarity* (names of objects: *rho =* – 0.33, *p* < 0.001; names of actions; *rho =* – 0.24, *p* < 0.001) and *Frequency* (names of objects: *rho =* – 0.4, *p* < 0.01; names of actions; *rho =* – 0.46, *p* < 0.01). Positive correlations were found between *Age of acquisition* and *Complexity index* (names of objects: *rho =* – 0.19, *p* < 0.001; names of actions: *rho =* – 0.32, *p* < 0.001).

*Frequency* showed significant negative correlations with *Complexity index* (*rho* = – 0.44, *p* < 0.001)*, Age of acquisition* (names of objects: *rho* = – 0.39, *p* < 0.001; names of actions; *rho =* – 0.54, *p* < 0.001) and *Naming latencies* of objects (*rho* = – 0.28, *p* < 0.001). For names of objects, *Frequency* was also positively correlated with *Concept familiarity* (*rho* = 0.19, *p* < 0.001).

Significant correlations were also found between *Image agreement* and *Concept familiarity* for pictures of actions (*rho* = 0.17, *p* < 0.001).

#### Predictors of naming latencies

We used linear mixed-effect models to explore which of the variables that correspond to the properties of pictures and their names are significant predictors of the pictures’ *Naming latencies.* The results of models fitted for the pictures of objects and pictures of actions are presented in Table 6. For the pictures representing objects, *Name agreement* (indexed by entropy (*H*)), *Age of acquisition*, *Frequency, Imageability, Word complexity,* and *Concept familiarity* turned out the be significant predictors of *Naming latencies*. For the pictures representing actions, we found that *Name agreement, Concept familiarity, Age of acquisition,* and *Frequency* significantly predicted *Naming latencies*.

**Table 6:** Results of the mixed-model analysis of the predictors of *Naming latencies* for CLT pictures representing objects and actions

	Estimate	Std. Error	*t* value	Effect size (d)
	Pictures of objects			
*Intercept*	*– 1.21*	*0.02*	*– 64.07*	
*(1) H*	– 0.31	0.05	– 6.59	– 1.04
*(2) Goodness of depiction*	0.01	0.09	0.09	0.01
*(3) Image agreement*	– 0.06	0.05	– 1.23	– 0.19
*(4) Concept familiarity*	– 0.01	0.01	– 2.22	– 0.35
*(5) Age of acquisition*	– 0.52	0.14	– 3.62	– 0.49
*(6) Imageability*	– 0.22	0.07	– 3.02	– 0.43
*(7) Frequency*	– 0.01	0.01	– 2.36	– 0.32
*(8) Complexity index*	– 0.03	0.01	– 2.18	– 0.33
	Pictures of actions			
*Intercept*	– 0.95	0.02	– 55.27	
*(1) H*	0.44	0.04	11.27	1.86
*(2) Goodness of depiction*	0.10	0.08	1.32	0.24
*(3) Image agreement*	– 0.06	0.04	– 1.55	– 0.28
*(4) Concept familiarity*	– 0.02	0.01	– 3.25	– 0.58
*(5) Age of acquisition*	– 0.29	0.13	– 2.21	– 0.29
*(6) Imageability*	0.09	0.07	1.31	0.16
*(7) Frequency*	0.01	0.00	2.38	0.31
*(8) Complexity index*	0.01	0.02	0.78	0.11

The analyses for pictures of actions are based on *Name agreement*, calculated based on aggregated and unified answers (Method 3 – for details see: *Scoring*).

Prior to the analyses, the positively skewed predictors of *H and Age of acquisition* were transformed using an inverse transformation to normalize their distribution. For this reason, the direction of these effects is inverted in the presented analysis (e.g., a *negative* effect of *Age of acquisition* reported in the table shows that pictures whose names are acquired *later* in life (higher *Age of acquisition*) correspond to slower naming latencies)

## Discussion

The aim of the current paper was to provide norms for the CLT pictures of objects and actions and their corresponding names in Polish. Our study contributes to the existing normed databases of pictures in three important ways: (1) it provides the first normed set of pictures for Polish; (2) it is the first database of colored pictures which contains pictures representing both objects and actions drawn in a comparable style; (3) the pictures in the CLT database were carefully prepared to provide a valid tool for multicultural comparisons. In the following section, we will discuss in more detail the results of our analyses in relation to these three aspects of our study.

The first contribution of the current study is that it provides norms for a picture database for a new language: Polish. With over 40 million speakers worldwide, Polish is, after Russian, the second most spoken Slavic language in the world; it is the 43rd most-spoken language in the world (Eberhard et al., 2021). While previous studies provided norms for *Age of acquisition*, *Imageability*, *Concreteness*, and a set of affective dimensions for large sets of Polish words (Imbir, 2015, 2016; Łuniewska et al., 2016, 2021), our study is the first to provide norms for pictures and their corresponding names. As previously discussed, providing norms for picture databases in new languages is of crucial relevance for psycholinguistic research as it is plausible to assume that at least some of the differences in pointing out, e.g., significant predictors of *Naming latencies* might be driven by cross-linguistic differences (Bonin et al., 2004; Sanfeliu & Fernandez, 1996). For a morphologically rich language like Polish, this concern is especially relevant in the case of verbs describing pictures of actions. In the only norming study of action pictures concerning another Slavic language, Russian (Akinina et al., 2015), the authors found that a significant portion of non-target responses were morphological derivations of the target names. In the case of our study, we observed that *Name agreement* was lower (corresponding to a lower score in %Name agreement and a higher score in entropy (*H*)) when it was calculated based on the first strategy (see Methods), which used the actual responses, non-unified for tense, aspect and reflexivity (see Table 8 in the Appendix A).^13^ Furthermore, a recent meta-analyses suggested that experience-related factors (i.e., how often people see or interact with the depicted objects) may influence lexical processing and are consequently reflected in *Naming latencies* and accuracy (Souza et al., 2020). As such, providing norms for a new Slavic language allows the evaluation of the extent to which the correlations between naming latencies and features of items (i.e., pictures and their names) observed for other languages also hold true for Polish.

The second contribution of our study is that it provides psycholinguistic norms for the first set of colored pictures of objects and actions. All three existing databases which provide pictures of objects and actions (i.e., the International Picture Naming project as well as the databases created by Khwaileh et al. (2018) and Bayram et al. (2017)) are based on black-and-white drawings. However, colored drawings have been shown to provide a much better depiction of actions than black-and-white drawings or photographs (Haman et al., 2015). An additional asset of the CLT pictures is that objects and actions are drawn in a comparable style following a similar detailed procedure (Haman et al., 2015). In this way, the CLT database can be a valuable tool in research looking at how grammatical class affects language processing (for a review of behavioral and neuroimaging findings, see: Vigliocco et al., 2011). Even though our cognitive system does not seem to organize conceptual and lexical knowledge by grammatical class (e.g., verbs vs. nouns; see Vigliocco et al., 2011; Vonk et al., 2019), there are still well-documented differences in how we process nouns and verbs and their corresponding concepts (Gentner, 1982, 2006; Gentner & Boroditsky, 2001). These differences have been proposed to reflect the varying difficulty or task demands associated with processing words pertaining to distinct grammatical classes. Differences in difficulty of processing of these two classes of words are related to the fact that verbs tend to be more morphologically complex and less imageable (Mätzig et al., 2009; for a discussion see: Khwaileh et al., 2018) or more abstract (Akinina et al., 2015; Vonk et al., 2019). To fully grasp the fine-grained differences in naming objects (using nouns) and actions (using verbs), it is crucial that the experimental stimuli be matched as closely as possible in respect to dimensions known to affect naming speed. An example is *Age of acquisition*, which has been found to be a significant predictor of naming speed in many studies, thus showing that later acquired words tend to be retrieved slower (e.g., Bonin et al., 2004; Cuetos & Alija, 2003; Dell’acqua et al., 2000; Liu et al., 2011; Székely et al., 2003; Székely et al., 2005; Weekes et al., 2007). At the same time, names of objects tend to be acquired earlier in life than names of actions (Gentner, 1982). As such, if not properly controlled, the effect of *Age of acquisition* can confound the observed differences in the processing of nouns and verbs because higher naming latencies for verbs could be related not only to differences in grammatical class but also to the fact that verbs are acquired later in life. Summing up, despite the challenges related to matching pictures of objects and actions (for a discussion of some of these challenges, see also: Székely et al., 2005), the good match between the pictures of objects and actions can be considered an additional asset of the CLT database.

Following two previous studies which compared characteristics of pictures of objects and actions (Bayram et al., 2017; Székely et al., 2005), we tested whether the CLT pictures of objects and actions differ with respect to any of the pictures’ properties and names. Similarly to the comparison of the IPNP action and object pictures as well as the comparison of the database of pictures by Bayram et al. (2017), our analysis revealed that pictures of objects are characterized by higher *Name agreement*, and they are named faster than pictures of actions. Importantly, in comparison to the IPNP pictures, the CLT pictures seem to have a higher *Name agreement* (for a comparison of other available databases of pictures and CLT pictures of objects, see Appendix Table 7; for pictures of actions, see Table 8 in the Appendix A, also available at osf.io/gp9qd). Moreover, in the IPNP study and the study by Bayram et al. (2017), significant differences were found for almost all dimensions that have been reported to describe pictures of objects and actions (incl. *Frequency*, *Age of acquisition*, *Word length,* and *Conceptual complexity* for the IPNP database and *Concept familiarity*, *Imageability*, *Word length*, *Morpheme count* and *Complexity* for database by Bayram et al., 2017). For the CLT pictures, we did not find significant differences that correspond to *Frequency, Age of acquisition* and *Complexity index* of the dominant names of pictures of objects vs. actions. Furthermore, no differences were found for *Image agreement* and *Concept familiarity*. We only found differences between pictures of objects vs. actions in their *Goodness of depiction* (pictures of objects were rated as representing the depicted objects better than pictures of actions) and *Imageability* (with nouns being easier to imagine than verbs). Summing up, the CLT database provides norms for pictures of objects and actions that are more closely matched than in any of the previous databases, which constitutes an additional asset for psycholinguistic research.

Finally, the third important aspect of the CLT database is related to the pictures for which norms are provided. More specifically, the procedure of creating the CLT pictures ensured cross-linguistic comparability by choosing pictures characterized by high *Name agreement* scores in multiple languages and by choosing the pictorial style that is valid and appropriate in multiple cultural contexts (for details of the procedure, see Haman et al., 2015, or footnote 5). This way of selecting the pictures makes CLT pictures a valid tool for experimental purposes in different languages as well as for cross-linguistic experiments (e.g., Altman et al., 2017; Bohnacker et al., 2016; Gatt et al., 2017; Haman et al., 2017; Hansen et al., 2019; Łuniewska et al., 2021; Potgieter & Southwood, 2016; Van Wonderen & Unsworth, 2020; Yap et al., 2017). The issue of cultural validity was also taken into account by Khwaileh et al. (2018): when these researchers created their database of pictures, they made sure that all pictures in their database are appropriate from the point of view of Gulf Arabic speakers. However, while this approach makes it possible to construct a highly valid tool for research carried out in a particular cultural context, it might bias the scope of the database to match the experiences of a specific group of speakers better than others. The CLT pictures circumvent the problem of cross-cultural validity in a more systematic way, although direct cross-cultural comparisons are beyond the scope of our paper. An additional asset of the CLT pictures with regards to cross-cultural validity is that – apart from the set of pictures used in the current norming study – there are additional variants of pictures (and new pictures and variants are being added). In the case of pictures of objects, they differ in color or minor details, but they also contain more and less modern variants of common items as well as regional variants of some of the objects. In the case of pictures of actions, the additional variants correspond to actors of different gender, age, and ethnicity. As such, future studies using CLT pictures for experimental research or providing norms for other languages should adjust their experiments to the cultural context by choosing the appropriate variants of pictures. However, even though the current norming study was run on adults, the CLT pictures were prepared for children and, as such, the choice of items might be limited as compared to other available databases.

### Correlations among the characteristics of object and action pictures – Comparison with previous studies

The correlational analyses yielded a similar pattern of results for CLT pictures representing objects and actions. Similarly to previous studies, we found significant negative correlations between *Age of acquisition* and *Imageability, Concept familiarity* and *Frequency* for pictures of objects (Alario et al., 2004; Bakhtiar et al., 2013; Moreno-Martínez & Montoro, 2012; Tsaparina et al., 2011; Weekes et al., 2007) and *Age of acquisition* and *Concept familiarity* and *Frequency* for pictures of actions (Akinina et al., 2015; Bonin et al., 2004; Cuetos & Alija, 2003; Schwitter et al., 2004; Shao et al., 2014). Similar correlations were also reported for a set of combined pictures of objects and actions by Bayram et al. (2017). This result indicates a pattern that is consistent across languages, i.e., words acquired earlier in life tend to be frequently used names that correspond to rich and easily retrievable semantic concepts. Additionally, *Age of acquisition* showed a significant positive correlation with the *Complexity index*, which indicates that more complex words tend to be acquired later than less complex ones. Altogether, *Age of acquisition* shows correlations with variables related to conceptual processing (*Imageability* and *Concept familiarity*), as well as variables related to phonological and morphological processing (*Frequency* and *Complexity index*).

#### Predictors of naming latencies

The effect of *Name agreement* on picture *Naming latencies* has been consistently reported in all previous norming studies which explored the predictors of *Naming latencies* in databases of object pictures (Alario et al., 2004; Bakhtiar et al., 2013; Bangalore et al., 2022; Barry et al., 1997; Cuetos et al., 1999; Dell’acqua et al., 2000; Khwaileh et al., 2018; Liu et al., 2011; Nishimoto et al., 2005; Severens et al., 2005; Shao & Stiegert, 2016; Székely et al., 2003; Torrance et al., 2018; Weekes et al., 2007) and action pictures (Bonin et al., 2004; Cuetos & Alija, 2003; Khwaileh et al., 2018; Schwitter et al., 2004; Shao et al., 2014; Székely et al., 2005). Our results confirm that *Name agreement* is also a robust predictor of *Naming latencies* in Polish for all CLT pictures.

We also found significant effects of *Frequency* and *Age of acquisition* for pictures of both objects and actions. The results we observed for pictures of objects seem to reflect the general tendencies reported in the literature, which often indicate *Frequency* and/or *Age of acquisition* as significant predictors of *Naming latencies.* However, there are discrepancies in reporting the effects of *Age of acquisition* and *Frequency* that have sparked a debate on the independence of these two effects (Brysbaert & Cortese, 2011; Brysbaert & Ghyselinck, 2006). Based on the correlation analyses reported in previous studies, we know that *Age of acquisition* and *Frequency* tend to be correlated: more frequently used words are usually acquired earlier in life. As such, whenever one of these effects is included in an analysis of *Naming latencies* predictors, it can influence the estimation of the other effect. In fact, in most norming studies that have taken the *Age of acquisition* and *Frequency* effects into account, only the *Age of acquisition* effect has been found to be a significant predictor of *Naming latencies* (Bangalore et al., 2022; Bonin et al., 2004; Cuetos & Alija, 2003; Dell’acqua et al., 2000; Liu et al., 2011; Székely et al., 2005; Székely et al., 2003; Weekes et al., 2007; for a review see: Juhasz, 2005). Independent modulation of *Naming latencies* by the effects of *Frequency* and *Age of acquisition* has been found in a few studies (Alario et al., 2004; Bakhtiar et al., 2013; Cuetos et al., 1999; Khwaileh et al., 2018). The effect of *Frequency* has also been found to be a significant predictor of *Naming latencies* when no *Age of acquisition* effect was observed; however, in these cases, *Age of acquisition* was either not included in the analyses (Bates et al., 2003), or some degree of collinearity was observed for the predictors of *Frequency* and *Age of acquisition* (VIF^14^ Frequency = 2.08; VIF *Age of acquisition* = 3.72; Shao & Stiegert, 2016). The collinearity problem has been identified as one of the most probable causes of the discrepancies between experiments testing the effects of *Age of acquisition* and *Frequency* on *Naming latencies* (Juhasz, 2005). However, it has also been argued that since the origin of the effects of *Age of acquisition* and *Frequency* on *Naming latencies* is different (for a discussion, see: Barry et al., 1997), it is possible to estimate the partially independent contributions of these two effects in a *Naming latencies* analysis (Székely et al., 2005). Importantly, *Age of acquisition* and *Frequency* can also interact with each other: it has been shown that the effect of *Frequency* is very pronounced for pictures whose names are acquired late, but it is absent for early acquired names (Barry et al., 1997). As such, the absence of the *Frequency* effect in some previous studies may also be related to insufficient ranges in *Age of acquisition* scores. However, the results of the current study indicate that the pictures of objects in the CLT database are not affected by the issues described above. We did not observe a high degree of multicollinearity: the *Frequency* and *Age of acquisition* of the names of CLT pictures were only moderately correlated (pictures of objects, *rho* = – 0.39; pictures of actions, *rho* = – 0.54).

As for pictures of actions, our results also align with previous findings. So far, all of the norming studies of action pictures have reported significant effects of *Age of acquisition* (Bonin et al., 2004; Cuetos & Alija, 2003; Schwitter et al., 2004; Shao et al., 2014; Székely et al., 2005). However, the *Frequency* effect for pictures of actions that we found in our analysis was previously found in only one norming study (Székely et al., 2005). Interestingly, similarly to the study of Székely et al., we found that the effect of *Frequency* for pictures of actions had an opposite direction compared to the effect for pictures of objects: higher-frequency names of actions took longer to produce than lower-frequency names. Székely et al. (2005) proposed that this effect could be driven by the fact that when speakers fail to find a more precise lower-frequency name for a picture of an action, they tend to use high-frequency multipurpose verbs (“light words”) as a replacement. However, it is unclear why this strategy would not be used in the case of nouns. One possibility is that names of objects are generally higher-frequency words that are more easily accessible; as such, speakers have no problems finding the appropriate names. Another possibility is that in the case of nouns it is difficult to find a multipurpose “light word” which could be used to describe an object on a more general level. As such, the effect of *Frequency* observed in *Naming latencies* of pictures of actions may in fact correspond to the naming strategy; it is not necessarily a direct index of the ease of lexical access, as is in the case of pictures of objects.

The last predictor of *Naming latencies* found in pictures of both objects and actions was *Concept familiarity*, significant effects of which were found in several previous norming studies (Bangalore et al., 2022; Cuetos et al., 1999; Nishimoto et al., 2005; Torrance et al., 2018; Weekes et al., 2007); however, the effect of *Concept familiarity* has not been consistently reported in the literature. Liu et al. (2011) have argued that *Concept familiarity* may in fact be closely related to the effect of *Frequency*; as such, its effect may be reduced when the effect of *Frequency* (and *Age of acquisition*) is explicitly controlled for. Even though in our study the effects of *Concept familiarity* and *Frequency* do not seem to be confounded for pictures of either objects or actions^15^, it seems plausible that the effect of *Concept familiarity* reflects the ease of conceptual retrieval and object recognition. First of all, the effect of *Concept familiarity* seems to affect the picture-naming process at earlier stages than *Age of acquisition* and *Frequency*, namely object (or action) recognition and the retrieval of semantic representations corresponding to a picture’s name (Alario et al., 2004). Snodgrass and Vanderwart (1980) argue that *Concept familiarity* can be seen as analogous to the *Frequency* effect, but instead of being related to a picture’s name it is related to its meaning. What this means is that while *Frequency* is driven by exposure to the word form, *Concept familiarity* reflects exposure to the concept itself – a kind of *concept frequency*, as opposed to word frequency (Khwaileh et al., 2018). This might explain why we do not observe significant effects of variables like *Imageability* or *Image agreement*, both of which are also thought to be related to the ease of conceptual processing; however, unlike *Concept familiarity*, they do not directly depend on how often the speaker has contact with a given concept^16^. Such an interpretation suggests that *Concept familiarity* is influenced to a larger degree by a speaker’s experience with objects and actions (without a direct relation with their corresponding names) than by the relationship between the concepts depicted in the pictures and their corresponding names, as in the case of *Imageability* and *Image agreement*.

While our results clearly show that *Naming latencies* of object and action pictures depend on the frequency of exposure to the concept they represent, it is unclear what the cause of the discrepancies between different studies that assessed *Concept familiarity* effects is. Liu et al. (2011) have argued that due to the close relationship between the effects of *Concept familiarity* and lexical *Frequency,* it is difficult to disentangle their influence on *Naming latencies*. As such, whenever *Frequency* is controlled for, no *Concept frequency* effect is observed. However, this explanation does not hold in the case of the CLT database: we observed the effect of *Concept familiarity* even though we controlled for the effect of *Frequency*. We propose that another possible explanation of the discrepancies in reporting the *Concept familiarity* effect is that it depends on the database of pictures used in a given study. Interestingly, with the exception of the study by Khwaileh et al. (2018), all experiments that found a significant effect of *Concept familiarity* on *Naming latencies* used stimuli from the database created by Snodgrass and Vanderwart (1980) or its colored version (Rossion & Pourtois, 2004). As such, it is plausible to assume that *Concept familiarity* is strongly bound to the characteristics of a given database of pictures. *Concept familiarity* is only a significant predictor of *Naming latencies* for databases that include a wide range of objects or actions that people see or think about more or less often. This characteristic of the CLT database could be an important asset for future studies exploring conceptual and semantic processing based on a picture-naming task.

We also found an effect of *Imageability* on *Naming latencies*; however, it was limited to pictures of objects. Similarly to previously reported results, we found that more imageable names were produced faster than less imageable ones (e.g., Alario et al., 2004; Bakhtiar et al., 2013; Shao & Stiegert, 2016). At the same time, the effect of *Imageability* has only been observed in one study on pictures of actions (Shao et al., 2014). This pattern of results is in line with previous studies that showed a stronger relationship between *Imageability* and *Naming latencies* in nouns than in verbs (Chiarello et al., 1999) and a general tendency for nouns to be more imaginable than verbs (Bird et al., 2001; Simonsen et al., 2013). Interestingly, it has been shown that when *Imageability* is controlled for, differences in how nouns and verbs are processed disappear (Vonk et al., 2019; for a review of behavioral and neuroimaging studies see Vigliocco et al., 2011). This was explained by referring to words’ concreteness, which is closely linked to *Imageability* (Vonk et al., 2019): concrete words are easier to imagine than abstract concepts. For example, in a lexical decision task it was shown that the effect of concreteness was only present for nouns: concrete nouns are processed faster than abstract ones (Vonk et al., 2019). On the other hand, the same study found that concreteness did not affect processing of verbs (Vonk et al., 2019). The results of our study extend these findings to a picture-naming task.

Finally, unlike some of the previous experiments, we did not find significant effects of *Image agreement* for the CLT pictures. As previously discussed, this measure reflects processes at the intersection of conceptual-semantic processing and object recognition. The fact that we did not find *Image agreement* to significantly predict naming latencies might suggest that the CLT pictures are based on a set of concepts that are easily identifiable and retrievable.

## Conclusion

In the current paper, we present Polish norms for a set of 164 colored pictures representing objects and 146 colored pictures representing actions: the CLT pictures. The CLT database is the first database of pictures of objects and actions and their corresponding names normed for Polish in terms of *Naming latencies, Name agreement, Goodness of depiction, Image agreement, Concept familiarity, Age of acquisition, Imageability.* In the current study, we also report estimates of *Lexical Frequency* and *Word complexity* which correspond to the names of the presented pictures. To the best of our knowledge, our contribution is the first normed set of colored pictures in which object and action pictures were created in the same style. A large set of psycholinguistic variables related to characteristics of pictures and their corresponding names was used in the study, thus comparison of Polish norms with existing data for other languages was possible. We have shown that CLT pictures are characterized by very high *Name agreement*, even when it is compared to the *Name agreement* obtained for databases of real object pictures (Brodeur et al., 2012, 2014; Moreno-Martínez & Montoro, 2012; Souza et al., 2021) that were created as a more ecologically valid alternative to the Snodgrass and Vanderwart (1980) database. We have also shown that the norms and estimates that correspond to the CLT pictures and names yield a similar pattern of correlations as those found in other languages and databases. Together with the fact that the CLT pictures were created to ensure that pictorial style is cross-culturally appropriate, this indicates that the CLT database can be used in studies on different languages. Finally, we provide analyses of *Naming latency* predictors which show that *Name agreement, Concept familiarity, Age of acquisition* and *Frequency* have a significant effect on *Naming latencies* for both objects and actions; moreover, *Imageability* is an additional predictor of *Naming latencies* for pictures of objects. We hope that the CLT database will become a valuable resource of experimental material for psycholinguistic studies using pictures of objects and actions, not only for Polish but also for other languages.

### Supplementary Information

Below is the link to the electronic supplementary material.

Supplementary file1 (XLSX 133 KB)

Supplementary file1 (XLSX 424 KB)

## Data Availability

All datasets analyzed in the current study as well as the norms corresponding to the CLT pictures of objects and actions are freely available in osf.io/gp9qd. The CLT database of pictures, along with its extensions (picture variants), is available on request on osf.io/y2cwr. The pictures are shared for free for research purposes. For any questions related to sharing and accessing the CLT pictures, please contact Magdalena Łuniewska (magdalena.luniewska@psych.uw.edu.pl). The pictures are not available in unlimited open access as they are a part of a diagnostic tool forming part of the LITMUS battery of tests, which is used to assess the comprehension and production of nouns and verbs in multilingual children. To ensure the validity of the diagnosis based on the CLTs, it is crucial to make sure the materials used in the tasks are not easily available to the general public.

## Appendix A

**Table 7:** Descriptive statistics for Name agreement norms (H statistic and Name agreement (%)) obtained for the pictures of **objects** used in the current study and a selection of other normative studies on different databases of pictures and languages

	Picture database	Language	*H statistic*			Name agreement (%)		
			Mean	SD	Range	Mean	SD	Range
Current study	CLT pictures	Polish	0.37	0.44	0–1.92	91.43%	13.18%	32.63–100 %
Bates et al. (2003)	International Picture Naming Project	Bulgarian	0.84	0.65	0–2.70	80.2%	20.4%	13–100%
		Chinese	1.16	0.79	0–3.57	71.9%	23.3%	11–100%
		English	0.67	0.61	0–2.90	85.0%	16.4%	28–100%
		German	0.76	0.68	0–3.28	81.1%	19.9%	21–100%
		Hungarian	0.91	0.73	0–3.52	78.0%	21.3%	13–100%
		Italian	0.95	0.73	0–3.47	77.0%	21.6%	12–100%
		Spanish	0.86	0.72	0–2.90	80.0%	20.4%	17–100%
Bayram et al. (2017)	Custom line drawings	Turkish	0.07	0.16	-	98.67%	3.17%	-
Brodeur et al. (2012) *	BOSS	English	1.65	1.01	0–4.1	64%	23%	20–100%
		French	1.56	0.97	0–3.83	63%	24%	14–100%
Clarke and Ludington (2018) *	BOSS	Thai	1.65	0.95	0–3.87	59.45%	24.89%	8–100%
Duñabeitia et al. (2018) *	MultiPic	Dutch (Belgium)	0.83	0.73		78.20%	20.90%	
		Dutch (Netherlands)	0.78	0.73		79.70%	20.70%	
		English	0.78	0.70		79.90%	20.20%	
		French	0.71	0.68		82.50%	18.90%	
		German	0.86	0.75		78.50%	20.80%	
		Italian	0.69	0.67		82.90%	18.60%	
		Spanish	0.50	0.58		87.60%	16.50%	
Khwaileh et al. (2018)	Custom line drawings	Gulf Arabic	-	-	-	86%	17%	40–100%^+^
Krautz and Keuleers (2021)	LinguaPix	German	0.69	0.70	-	79%	23%	-
Moreno-Martínez and Montoro (2012) *	Photographs of real objects	Spanish	0.94	0.87	0–3.73	72%	28%	1–100%
Rossion and Pourtois (2004)	S & V (line drawings)	French	0.44	0.56	0–2.44	88.2%	17.10%	20–100%
	S & V (colored)	French	0.32	0.46	0–1.65	90.3%	16.90%	39–100%
Souza et al. (2021) *	RealPic	Portuguese	0.78	0.90	0–5.58	77.94%	20.97%	21–100%
Torrance et al. (2018) *	S & V (colored)	Bulgarian	0.76	0.80	0–3.96	83.20%	19.60%	18.4–100%
		Dutch	0.72	0.70	0–3.16	83.00%	19.10%	20.3–100%
		English	0.66	0.75	0–4.21	85.00%	18.90%	17–100%
		Finnish	0.83	0.79	0–3.92	80.40%	20.90%	18–100%
		French	0.75	0.68	0–3.84	83.60%	18.30%	17.9–100%
		German	0.67	0.72	0–4.16	84.30%	18.70%	14.3–100%
		Greek	0.82	0.78	0–3.22	83.10%	18.60%	19.8–100%
		Icelandic	0.75	0.74	0–3.5	82.50%	20.00%	19.4–100%
		Italian	0.65	0.68	0–3.48	84.90%	18.40%	24.1–100%
		Norwegian	0.75	0.80	0–3.75	82.90%	20.10%	23.5–100%
		Portuguese	0.74	0.84	0–3.76	83.80%	20.10%	22.7–100%
		Russian	0.80	0.79	0–3.98	81.90%	20.20%	14.4–100%
		Spanish	0.70	0.80	0–3.68	85.20%	19.10%	21–100%
		Swedish	0.58	0.68	0–2.99	86.30%	18.30%	21.7–100%
Tsaparina et al. (2011)	S & V (colored)	Russian	0.82	0.73	0–3.71	80.63%	19.64%	17.39–100%
Yoon et al. (2004) *	S & V (line drawings)	American English	0.57	0.60	0–3	87%	16%	28–100%
		Chinese	1.41	1.74	0–4.67	67%	25%	0–100%

* Name agreement norms based on written responses

^+^Name agreement was calculated based on filtered data with one criterion set to *Name agreement* score of 40% of higher.

S & V (line drawings) – database created by Snodgrass and Vanderwart (1980); S & V (colored) – colored version of the database created by Snodgrass and Vanderwart (1987) introduced by Rossion and Pourtois (2004)

**Table 8:** Descriptive statistics for Name agreement norms (H statistic and % Name agreement) obtained for pictures of **actions** of the current study and a selection of other normative studies on different databases of pictures and languages

	Picture database	Language	*H statistic*			Name agreement (%)		
			Mean	SD	Range	Mean	SD	Range
**Current study:** Actual answers	**CLT pictures**	Polish	1.06	0.75	0–3.57	75.37%	20.42%	21–100%
**Current study:** Recoded infinitives			1	0.72	0–3.25	76.71%	20.10%	24–100%
**Current study:** Unified infinitives			0.89	0.67	0–2.94	79.39%	18.80%	26–100%
Akinina et al. (2015)	Custom line drawings	Russian	1.2	0.76	0–3.66	76.01%	18.18%	28–100%
Bayram et al. (2017)	Custom line drawings	Turkish	0.28	0.28		94.26%	5.65%	
Bonin et al. (2004)	F & T	French	0.96	0.72	0–3.01	73%	21.51%	10–100%
Cuetos and Alija (2003)	M & D	Spanish	0.74	0.80	0–2.78	83.58%	21.29%	24–100%
Khwaileh et al. (2018)	Custom line drawings	Gulf Arabic				73%	21%	40–100%^+^
Shao et al. (2014)	M & D + K & M	Dutch	0.32	0.53	0–3.09	86%	14%	45–100%
Székely et al. (2005)	IPNP	English	1.2	0.77	0–2.88	71.30%	-	21–100%

^+^Name agreement was calculated based on filtered data with one criterion set to *Name agreement* score of 40% of higher.

D & M – database created by Masterson and Druks (1998); F & T – database created by Fiez and Tranel (1997); K & M – subset of 24 pictures created for the purposes of Konopka & Meyer (2014); IPNP – International Picture Naming Project set introduced in Székely et al. (2005). The three calculations of *Name agreement* in the CLT database correspond to three different ways of coding the participants’ answers, described in detail in *Methods*

## Footnotes

1 Experiments on emotional processing often use datasets of pictures normed for affective dimensions, such as the NAPS (Marchewka et al., 2014; Riegel et al., 2016) or IAPS datasets (Lang et al., 1993), which provide affective ratings corresponding to pictures (e.g., valence, emotional arousal, approach-avoidance etc.).

2 Psycholinguistic characteristics of words, such as *Age of acquisition*, *Imageability, Frequency* and many others, can also be found in databases focused on a large number of words in a given language, e.g., Peti-Stantić et al., 2021 (6000 words in Croatian); Imbir, 2016 (4900 words in Polish); Bird et al., 2001 (2645 words in British English); Simonsen et al., 2013 (1599 words in Norwegian) or by comparing words from a variety of different languages (Łuniewska et al., 2016, 2019)

3 The distinction between the characteristics of names and pictures is not always straightforward. For the purpose of this paper, we assumed that the characteristics of pictures refer to those variables which take one value for each picture. Characteristics of names, on the other hand, refer to variables which have a different value for each dominant or alternative name of a given picture; as such, they can have multiple levels for each picture. For example, Goodness of depiction is a characteristic of a picture because each picture has one corresponding score on this dimension; Lexical frequency, on the other hand, is a characteristic of the name of a picture because each picture can have multiple scores on this dimension that correspond to all its alternative names.

4 Both objective and subjective estimates of *Age of acquisition* were shown to be correlated (e.g., French: *r* = 0.687, Chalard et al., 2003; English: *r* = 0.93, Gilhooly & Gilhooly, 1980; Chinese: *r* = 0.502, Liu et al., 2011). While recent studies point out that objective *Age of acquisition* is actually a better predictor of naming latencies than subjective *Age of acquisition* (Chalard et al., 2003; Liu et al., 2011), both types of *Age of acquisition* affect naming latencies in the same direction: words learnt earlier in life are produced faster than words learnt later in life.

5 The creation procedure of the pictures was based on a cross-linguistic picture naming study which involved 1024 pictures from eight picture databases named by speakers of 34 languages. The databases of pictures tested in the process of creating the CLT pictures included sets of photographs from the BOSS dataset (Brodeur et al., 2012) and other databases, coloured pictures from Rossion and Pourtois’ (2004) set, and several other databases (pencil drawings, watercolors, with and without background), as well as black-and-white drawings (IPNP, Székely et al., 2005). The cross-linguistic picture naming study enabled the selection of a list of words corresponding to pictures that constituted the most valid set for the creation of a diagnostic tool for multiple different languages. Coloured drawings without background were assessed as the most suitable pictorial style for both pictures of objects and actions. The pictures in the CLT database were drawn on the basis of pictures named with the highest agreement across languages in the style that was assessed as the most suitable in all languages (Haman et al., 2015).

6 The scale used in the current study followed the one proposed by Haman et al., 2015. Even though coding with lower values that correspond to better *Goodness of depiction* might seem unnatural, we decided to keep the coding similar to the original study for better comparability. Importantly, participants selected the responses from a *list of predefined answers* without information on how they were coded, so zero for “very good” would not be misleading for them (i.e., they were familiar with the labels but not with the numerical values assigned to the responses).

7 The criteria for selection of words for the additional *Age of acquisition* rating study was driven pragmatically: for all pictures of objects, a total of 436 different names (including the dominant and alternative names) and for all pictures of verbs a total of 823 different names were given. It would be very challenging to collect norms for all names used by participants which were used in the picture naming study; hence, we decided to set a cut-off criterion which would allow us to collect norms for the most often used alternative names. Altogether, the norms derived from the previous study and the complementary *Age of acquisition* rating study corresponded to 72.1% of names used to name objects and 61% of names used to name actions.

8 We came up with this procedure by comparing different methods of determining the naming latencies: a method based on the voice key with threshold specified in dB, another method based on Chronset; an automated tool for detecting speech onset (Roux et al., 2017); and the manual determination of the naming times. The naming times determined using the voice key included a significant number of errors (e.g., naming times corresponded to a noise or a grunt preceding the answer), and they were also sensitive to the quality of the recordings, with clear and loud responses corresponding to much more accurate response time estimates than quiet or noisy recordings. Chronset made it possible to overcome most of the issues that affected the voice key method as it is specifically trained to recognize human speech. However, as the tool has not been validated or tested on Polish sounds, in some cases the estimated naming times were delayed or less accurate compared to the actual response time. Even though these delays were quite systematic and as such would not be a problem for an experimental study, in a norming study it is crucial that the response times accurately reflect the speech onset. The manual coding of the naming latencies allowed us to achieve this goal.

9 As pointed out by Krautz and Keuleers (2021), the value of *H* increases with the number of alternatives, which is largely driven by the number of participants. To circumvent this problem, Krautz and Keuleers (2021) proposed using a normalized entropy score which corrects for the number of alternative responses. We have included a normalized entropy measure in the Excel file that contains a full set of norms for the CLT database, which is available at osf.io/gp9qd as well as in the Supplementary Materials. However, to keep our study comparable to previous norming studies, we used the non-normalized entropy (*H*) score for the statistical analyses.

10 For example, following the 1st strategy, the following answers would be considered to be three alternative names: *biją się (to fight, 3*^*rd*^ *person, plural, reflexive form), bije (3*^*rd*^ *person, singular, non-reflexive form)* and *bije się (3*^*rd*^ *person, singular, reflexive form)*. Following the 2^nd^ strategy, the answers *biją się* and *bije się* would be recoded to the infinitive form *bić się (to fight, reflexive form)* and *bije* would be recoded to the infinitive form *bić (to fight, non-reflexive form)*, *thus* yielding two alternative names in total. Following the 3^rd^ strategy, all four answers would be considered to be one name – *bić się (to fight, reflexive form)* – corresponding to the infinitive form that was most frequently used by the participants of the picture naming study.

11 While the VIF scores for %*Name agreement* and *H score* for pictures of actions can be considered acceptable, we decided to drop the predictor of %*Name agreement* from both analyses to keep them analogous.

12 In the main text, we only discuss the models that represent the main effects of our predictors. However, we also ran exploratory analyses to test the effects of two-way interactions. As these analyses are not central for the norming study and they did not test any specific *a priori* hypotheses, we do not report them in this paper. Instead, they are described in more detail in a short report that can be found on OSF: osf.io/gp9qd.

13 Non-Slavic languages also allow a variety of morphological forms of a verb corresponding to the conjugated forms or different grammatical tenses. However, compared to other languages for which there are available norms for pictures of actions (i.e., English, French, and Spanish), a fundamental difference between Polish and Russian relates to the grammatical aspect: in Slavic languages, aspect is a grammatical category superior in relation to, e.g., tense; however, in English, French and Spanish, aspect is reflected by grammatical tense. A consequence of this is that Slavic languages have different *forms* of verbs corresponding to different aspects, which can be formed by either adding prefixes or altering the roots of the verbs. The inconsistencies in aspect were the main factor affecting the *Name agreement* scores of pictures of actions in the current study. For example, in the case of a picture representing the action of horse riding, 50.52% of alternative names (LexCode2) corresponded to the morphological variants of the dominant name (“jeździć” / “to ride”).

14 The variance inflation factor (VIF) is a measure of the amount of multicollinearity in a set of multiple regression variables. It is equal to the ratio of the overall model variance to the variance of a model that includes only that single independent variable. VIF values over 5–10 are considered to be indicative of high multicollinearity (for a discussion of the cut-off points, see: Craney & Surles, 2002). VIF values around 1 indicate that there is no multicollinearity between the predictors of a multiple regression model.

15 This statement is supported by the low correlations that we found between these two variables: *rho =* 0.19, *p* < 0.01 for pictures of objects and *rho =* 0.00, *p* = 0.46 for pictures of actions.

16 Interestingly, Khwaileh et al. (2018) found the effects of *Image agreement* on *Naming latencies* of both objects and actions; at the same time, *Concept familiarity* was not a significant predictor of action *Naming latencies*.

## Code availability

The code necessary to reproduce all the statistical analyses and figures reported in the current study is freely available on osf.io/gp9qd.
